# Evaluation of an Ultrafiltration-Based Procedure for Simultaneous Recovery of Diverse Microbes in Source Waters

**DOI:** 10.3390/w7031202

**Published:** 2015-03-18

**Authors:** Amy M. Kahler, Trisha B. Johnson, Donghyun Hahn, Jothikumar Narayanan, Gordana Derado, Vincent R. Hill

**Affiliations:** 1 Division of Foodborne, Waterborne, and Environmental Diseases, National Center for Emerging and Zoonotic Infectious Diseases, Centers for Disease Control and Prevention, Atlanta, GA 30329, USA; 2 Atlanta Research and Education Foundation, Decatur, GA 30033, USA

**Keywords:** groundwater, surface water, ultrafiltration, pathogen detection, Centricon Plus-70, polyethylene glycol, immunomagnetic separation (IMS)

## Abstract

In this study, hollow-fiber ultrafiltration (UF) was assessed for recovery of *Escherichia coli*, *Clostridium perfringens* spores, *Cryptosporidium parvum* oocysts, echovirus 1, and bacteriophages MS2 and ΦX174 from ground and surface waters. Microbes were seeded into twenty-two 50-L water samples that were collected from the Southeastern United States and concentrated to ∼500 mL by UF. Secondary concentration was performed for *C. parvum* by centrifugation followed by immunomagnetic separation. Secondary concentration for viruses was performed using centrifugal ultrafilters or polyethylene glycol precipitation. Nine water quality parameters were measured in each water sample to determine whether water quality data correlated with UF and secondary concentration recovery efficiencies. Average UF recovery efficiencies were 66%–95% for the six enteric microbes. Average recovery efficiencies for the secondary concentration methods were 35%–95% for *C. parvum* and the viruses. Overall, measured water quality parameters were not significantly associated with UF recovery efficiencies. However, recovery of ΦX174 was negatively correlated with turbidity. The recovery data demonstrate that UF can be an effective method for concentrating diverse microbes from ground and surface waters. This study highlights the utility of tangential-flow hollow fiber ultrafiltration for recovery of bacteria, viruses, and parasites from large volume environmental water samples.

## 1. Introduction

Unlike fecal indicator microorganisms (e.g., *Escherichia coli*), enteric pathogens causing waterborne diseases are often present in low concentrations in source water and drinking water. Traditional detection methods for waterborne pathogens, such as membrane filtration and U.S. Environmental Protection Agency (USEPA) Method 1623 and VIRADEL techniques, are either limited by the volume of water that can be processed, have low recovery rates, or can only be used to recover one microbe type [1–4]. Hollow-fiber ultrafiltration (UF) is a technique that allows for simultaneous concentration of multiple classes of microbes in large volume water samples. The method was first introduced in the 1970s as a means of concentrating viruses in water [5,6], but in recent years it has proven to be a robust technique for recovery of bacteria, viruses, and parasites in drinking and source water [7–22]. It has been reported to yield similar or higher recovery efficiencies than Method 1623 and VIRADEL for parasites and viruses in drinking water [1,17,23]. Despite having smaller pore sizes relative to microfilters, tangential-flow UF has the potential for application to low quality water because the method does not rely on capturing microbes in the filter, which could also trap other components of the water matrix and contribute to filter clogging. Microbes and other particles tend to remain in the bulk water sample as it is concentrated, due to the recirculation and scouring effect of the tangential-flow UF technique. Mull and Hill [19] performed UF using 40 liters of lake water with an average turbidity of 18 nephelometric turbidity units (NTU). Kuhn and Oshima [17] filtered 10 liters of surface water with turbidities up to 226 NTU.

Although UF is able to process large volumes of water, many components of the water matrix are co-concentrated during the UF procedure. This may affect microbe recovery from the UF procedure or downstream processing methods, as well as culture and non-culture detection assays. Humic and fulvic acids are known to inhibit PCR [24], and other water quality conditions such as pH, iron, and turbidity, may affect the performance of immunological techniques [25–27]. The role of water quality on UF performance and downstream processing and detection methods is not well understood. Turbidity is often the only measured water quality parameter during UF recovery studies, but few correlations have been made between turbidity and UF or total-method recovery efficiency [9,16–18]. Recent studies have begun to examine the relationship between multiple water quality parameters and UF recovery in raw ground water and finished drinking water [9,12].

In the present study, the effectiveness of tangential-flow hollow-fiber UF was evaluated for the recovery of six enteric pathogens and pathogen surrogates from 50-liter ground and surface water samples. The suite of microbes were chosen to represent multiple microbe classes and included *E. coli*, *Clostridium perfringens* spores, *Cryptosporidium parvum* oocysts, echovirus 1 (E1), and MS2 and ΦX174 bacteriophages. Secondary sample processing methods were also evaluated for recovery of *C. parvum*, E1, and the bacteriophages. Ground and surface water from Georgia and Tennessee (USA) were chosen to represent diverse water quality characteristics. Recovery efficiencies were compared to water quality parameters to determine the effect of water quality on method performance.

## 2. Materials and Methods

### 2.1. Water Samples

Ground and surface water samples were collected from 5 different sites in Georgia and Tennessee (USA). Surface water was obtained from the Chattahoochee River, Murphy Candler Park Lake, and Lake Allatoona in Atlanta, Georgia. Ground water was obtained from Lawrenceville, Georgia (artesian well, Piedmont Aquifer) and Jefferson City, Tennessee (Valley and Ridge aquifers, ground water under the influence of surface water). Water was collected from the Chattahoochee River on three different occasions to obtain samples with differing turbidity levels. Three to six 50-L water samples were collected from each site. Water samples were collected and transported to CDC at ambient temperature the day of collection or shipped priority overnight from Tennessee. Samples were held at 4 °C, but were allowed to equilibrate to ambient temperature overnight before an experiment. Before each experiment 50 L of water was poured into a sanitized 30-gallon high-density polyethylene tank.

### 2.2. Water Quality Testing

Water quality parameters were measured at CDC. pH was measured with a Fisher Scientific Accumet Research AR25 pH/mV/°C/ISE meter. Turbidity was measured with a Hach model 2100N turbidimeter. Specific conductance (SC) was measured with an Oakton CON 100 conductivity/°C meter. Total hardness measured using a Hach hardness test kit and AL-DT digital titrator. Alkalinity was measured using a Hach alkalinity test kit and AL-DT digital titrator. Total iron was measured with a Hach DR/2400 spectrophotometer and FerroVer iron reagent. Total organic carbon (TOC) and dissolved organic carbon (DOC) were measured using a Hach TOC reagent set and Hach DR/2400 spectrophotometer. Water samples were prepared for DOC analysis as described previously [12]. Total suspended solids (TSS) were measured according to Methods 2540D in *Standard Methods for the Examination of Water and Wastewater* [28].

### 2.3. Microorganisms and Microbial Assays

Six microbes were used in this study: bacteriophages MS2 and ΦX174, *E. coli*, *C. perfringens* spores, echovirus 1, and *C. parvum* oocysts. High seeding levels were used to allow for direct detection of the study microbes in the UF concentrate. MS2 and ΦX174 were produced and enumerated as described previously [12]. Background levels of MS2 and ΦX174 in the water samples plus the seeded amount resulted in a total input of 5810 ± 2680 and 15,100 ± 8990 PFU, respectively, per experiment. Echovirus 1 (E1) was propagated and enumerated in BGM cells as described previously [11]. Frozen stocks of E1 were diluted and filtered in the same manner as the bacteriophages and seeded at a level of 139,000 ± 97,800 PFU per experiment. Naturally occurring *E. coli* in each water sample was analyzed instead of seeded *E. coli*. Background levels of *E. coli* resulted in “input” levels of 26,900 ± 56,500 CFU per experiment. *E. coli* was enumerated by membrane filtration according to Methods 9222D and 9222G in *Standard Methods for the Examination of Water and Wastewater* [28]. *Clostridium perfringens* spores were purchased as BioBalls (BTF Pty. Ltd., Sydney, Australia). BioBalls were reconstituted as described previously [12] and passed through a 5-μm filter before seeding. Background levels of *C. perfringens* plus BioBall amounts resulted in input levels of 38,100 ± 85,600 CFU per experiment and were enumerated by membrane filtration using mCP agar [29]. *Cryptosporidium* oocysts were seeded directly from a refrigerated stock solution (Waterborne, New Orleans, LA, USA). Background levels of *C. parvum* in the water samples plus the seeded amount resulted in a total input of 5660 ± 7900 oocysts per experiment. *C. parvum oocysts* were enumerated by IFA microscopy as described in USEPA Method 1623 [30].

### 2.4. Ultrafilter Blocking

Ultrafilters were pre-treated with calf serum to minimize the adsorption of microbes. Five hundred milliliters of 5% calf serum (filter sterilized, Invitrogen No. 16170-078) was recirculated through the filter for 5 min with the filtrate port closed. The calf serum was then allowed to contact the ultrafilter fibers overnight at room temperature by rotating in an unheated hybridization oven. The calf serum was flushed from the ultrafilter before each experiment using 1 L of DI water.

### 2.5. Ultrafiltration Setup

The filtration components were set up as shown in Hill *et al*. [12]. A new Fresenius F200NR dialysis filter was used for each experiment (Fresenius Medical Care, Lexington, MA, USA). These hollow-fiber ultrafilters contain 2.0 m^2^ polysulfone hollow fibers having a ∼30,000 Dalton molecular weight cutoff. Platinum-cured L/S 36, L/S 24, and L/S 15 silicon tubing (Masterflex; Cole-Parmer Instrument Co., Vernon Hills, IL, USA) was used for each experiment. The 1-L HDPE bottle and all tubing connectors and clamps were autoclaved prior to each experiment. A syringe pump was used to inject the seeded microbes into the recirculation tubing. The ultrafilter, tubing, and syringe were discarded after each experiment. A Cole-Parmer Model 7550-30 pump drive and a Cole-Parmer Model 77201-62 peristaltic pump were used for all experiments.

### 2.6. Ultrafiltration Procedure

Before each UF procedure, 0.01% sodium polyphosphate [NaPP (Sigma-Aldrich No. 305553)] was added to the 50-L water sample and mixed. Sample water was pumped at a nominal rate of 2900 mL/min. The system was operated at 13 ± 2 psi to achieve a filtrate rate of ∼1200 mL/min and corresponding cross-flow rate within the ultrafilter of ∼1700 mL/min. Filtration was performed until ∼250 mL of concentrated sample remained in the recirculation loop consisting of the 1-L bottle, ultrafilter, and tubing. Using a 3-way stopcock, effluent flow from the 1-L bottle was closed off and the peristaltic pump was used to force as much of the retentate as possible into the 1-L bottle. The 1-L bottle was disconnected entirely and a bottle containing 500 mL of an eluent solution (0.01% Tween 80, 0.01% NaPP, 0.001% Antifoam Y-30 Emulsion) was connected in its place. The eluent was recirculated through the ultrafilter until air bubbles began entering the tubing. The inlet tubing was then pulled from the eluent bottle to allow air to flush remaining eluent from the system. The collected eluent (200–250 mL) was added to the retentate sample to produce a final concentrated sample for analysis or secondary processing. The average UF concentrate volume achieved using this protocol was 490 ± 50 mL.

### 2.7. Secondary Sample Processing Procedures

Centrifugal UF and polyethylene glycol (PEG) precipitation were performed to concentrate the primary hollow fiber UF concentrate for viral plaque assays. Two Centricon Plus-70 units (Millipore, Billerica, MA, USA) were used to concentrate 120 mL UF concentrate. The procedure was conducted according to the manufacturer's instructions, except a pre-rinse was not performed and the spin time was increased as needed to process the entire volume. The retentates recovered from each Centricon unit were combined to form one retentate. Particulate matter still attached to the cup surface after recovery of the retentate was rinsed from the filter with the modified PBS diluent and the extra volume was added to the retentate. The average Centricon concentrate volume was 8.8 ± 3.5 mL. PEG was used to concentrate 150 mL UF concentrate. A 10% bovine serum albumin (BSA) solution was added to the concentrates to achieve a final concentration of 1% BSA, followed by sequential addition of 0.9 M NaCl and 12% PEG. The pH was adjusted as needed to 7.0–7.4 with dilute HCl or NaOH and the amended concentrates were held for 1 h at 4 °C. The concentrates were centrifuged at 10,000 × *g* for 30 min (4 °C) and the PEG pellet was resuspended with the modified PBS diluent. The average PEG concentrate volume was 6.6 ± 1.3 mL. Centricon and PEG concentrates were assayed for MS2, ΦX174, and E1 by their respective plaque assays.

A 40-mL volume of UF concentrate was processed by centrifugation and immunomagnetic separation (IMS) to recover *C. parvum* oocysts. UF concentrates were centrifuged at 4000 × *g* for 30 min (4 °C). All but 5 mL of the supernatant and pellet volume was aspirated off and the remaining 5 mL was processed by IMS using an Aureon Crypto kit (Aureon Biosystems, Vienna, Austria). IMS was conducted according to manufacturer's directions except acid dissociation was done with 0.1 N HCl instead of 2-mercaptoethanol. IMS-processed samples were examined by the IFA microscopy procedure using an Easy-Stain kit (BTF, Sydney, Australia).

### 2.8. Data Analysis and Statistics

Recovery efficiencies, expressed as percentages, were calculated by dividing the number of microbes recovered after each procedure (concentration × sample volume) by the number of experimentally determined microbes that were present prior to the procedure (concentration × sample volume) and multiplying the result by 100. Total method recovery efficiencies for UF and secondary concentration were calculated by multiplying the two recovery efficiencies. Coefficient of variation (COV) was calculated by dividing the standard deviation by the respective mean recovery efficiency.

Pearson and Spearman correlation coefficients were used to assess the associations between water quality parameters and method recovery efficiency. A Wilcoxon signed-rank test was used to compare the recovery efficiencies of the PEG and Centricon procedures. To determine whether UF recovery efficiency varied by microbe, a one-way fixed effects analysis of variance (ANOVA) was used. The Tukey-Kramer HSD post hoc test was then used to perform a pairwise comparison between mean recovery efficiency of two microbes.

## 3. Results and Discussion

### 3.1. Water Quality

The pH for the water samples ranged from 5.4 in the Chattahoochee River to 9.6 in Lake Allatoona (Table 1). Water samples used in this study had a wide range of turbidity values, and ranged from 0.1 NTU in Lawrenceville ground water to 128 NTU in the Chattahoochee River. Specific conductance ranged from 53 μS/cm in the Chattahoochee River to 693 μS/cm in Jefferson City ground water. Hardness and alkalinity values were similar, ranging from 14 to 16 mg/L in the Chattahoochee River to 250–298 mg/L in Jefferson City ground water. Total iron ranged from <0.02 mg/L in Jefferson City ground water to 1.6 mg/L in the Chattahoochee River. TOC and DOC testing indicated that most organic carbon was present as dissolved compounds, with DOC ranging from 0.9 mg/L in Lake Allatoona to 28 mg/L in Jefferson City ground water. TSS ranged from 0.15 mg/L in Lawrenceville ground water to 57 mg/L in the Chattahoochee River. Turbidity was positively correlated with TSS (*R*^2^ = 0.97), but there was no correlation between turbidity and TOC or DOC. Ground water samples had the highest alkalinity, specific conductance, and hardness and the lowest turbidity and TSS. Jefferson City ground water had the highest TOC and DOC, and the lowest iron levels. Surface water samples had the highest turbidity and TSS levels. The Chattahoochee River had the lowest specific conductance, hardness, alkalinity, TOC, and DOC.

### 3.2. Microbial Recovery by ultrafiltration (UF)

**Figure 1:**
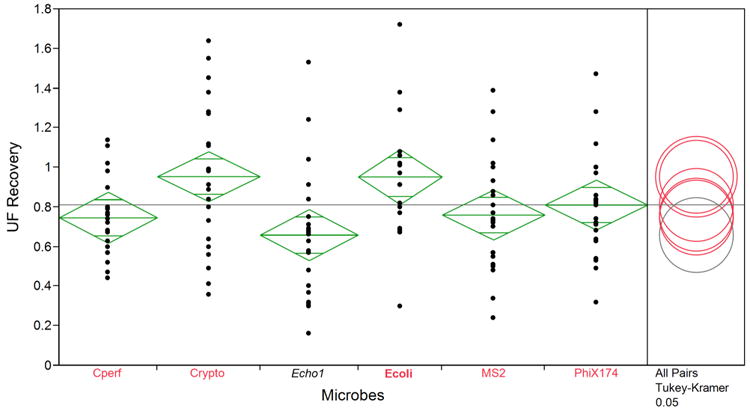
One-way ANOVA for UF percent recovery. Diamonds represent means (line near the center of each diamond), with 95% confidence intervals for each mean (the vertical span), based on the pooled estimate of the standard error. Comparison circles summarize the results of the multiple comparison procedure. The selected mean has bold, red circle and variable label (in this Figure, *E. coli*). Means that are not significantly different from the selected mean have unbolded, red circles and variable labels. Means that are significantly different from the selected mean have gray circles and gray italicized variable labels. In this example, the mean for *E. coli* is significantly different from the mean for echovirus 1, but is not significantly different from the mean for other microbes.

Across all water types, average recovery efficiencies for the UF procedure ranged from 66% for E1 to 95% for *C. parvum* (Table 2). Despite the high overall recovery efficiencies, the COVs for all microbes were also quite high and ranged from 26% (*E. coli* and *C. perfringens*) to 50% (E1). For some experiments, recovery efficiencies for the study microbes were >100%. This may have been due to aggregation of the microbe stocks, despite attempts to minimize aggregates by using the modified PBS diluent containing Tween 80, pre-filtering the seeding dilution, and vigorously shaking the *C. perfringens* BioBall seed suspension. Although the overall *F* statistics from one-way ANOVA (*F* = 3.2327, *p* = 0.009) suggested that the mean recovery rates were not equal across microbes, based on Tukey-Kramer pairwise comparisons, there were not many differences in UF recovery efficiency by microbe. The only exception was for E1 and *C. parvum* (*p* = 0.0134), for which the highest and lowest recovery efficiencies were observed (Figure 1).

### 3.3. Microbial Recovery by Secondary Concentration

Recovery efficiencies for the secondary concentration procedures are shown in Table 3. Average recovery efficiencies for the Centricon procedure for MS2, ΦX174, and E1 were 79%, 70%, and 35%, respectively. Total method recovery efficiencies, including UF and Centricon processing, averaged 61%, 58%, and 22% for MS2, ΦX174, and E1, respectively (Table A1). Average recovery efficiencies for the PEG procedure for MS2, ΦX174, and E1 were 78%, 76%, and 92%, respectively. Total method recovery efficiencies, including UF and PEG precipitation processing, averaged 59%, 61%, and 51% for MS2, ΦX174, and E1, respectively (Table A1). None of the measured water quality parameters were significantly associated with MS2 and ΦX174 recovery efficiencies. Recovery efficiencies for E1 with the Centricon and PEG procedures were negatively correlated with turbidity, although the association was not statistically significant [corr = −0.3821, 95% CI = (−0.6922, 0.0471) and corr = −0.4004, 95% CI = (−0.7033, 0.0255)]. The PEG procedure resulted in significantly higher recoveries of E1 than Centricon procedure (*p* = 0.0016).

## 4. Discussion

The results of this study demonstrate that tangential-flow hollow-fiber UF can be an effective technique for recovering diverse microbes from surface water samples. Recovery data reported for this study are similar to a recent study investigating hollow-fiber UF recovery in 100-liter surface water samples, where recovery rates of *E. co*li and MS2 from low seed experiments were 71% and 84%, respectively [10]. In the present study, E1 was the analyte with the lowest average recovery efficiency (66%, Figure 1), however this average recovery was relatively higher than the average recovery of poliovirus (40%) reported by Gibson *et al*. [10]. *C. perfringens* recovery was reported to be 30% by Gibson *et al*., which is lower than the 75% recovery reported here. However, this could be due in part to the fact that the *C. perfringens* evaluated in this study was naturally occurring or freeze dried in the form of a BioBall, whereas the *C. perfringens* used by Gibson *et al*., was laboratory-grown. Another study investigating hollow-fiber UF for simultaneous recovery of diverse microbes reported recovery efficiencies of 87%–96% for *E. coli* from 10-L ground and surface water samples, which is similar to the *E. coli* recovery efficiencies measured in the present study [18]. Morales-Morales *et al*., did not observe a difference in UF performance for recovery of *E. coli* in 0.3 NTU and 29 NTU water. In another tangential-flow UF study, *C. parvum* oocysts were recovered from 2-L ground and surface water samples with efficiencies ranging from 75% to 81% [16]. Although turbidity values in that study ranged from 0.3 to 31 NTU, there was no observed association between turbidity and recovery efficiency.

**Figure 2:**
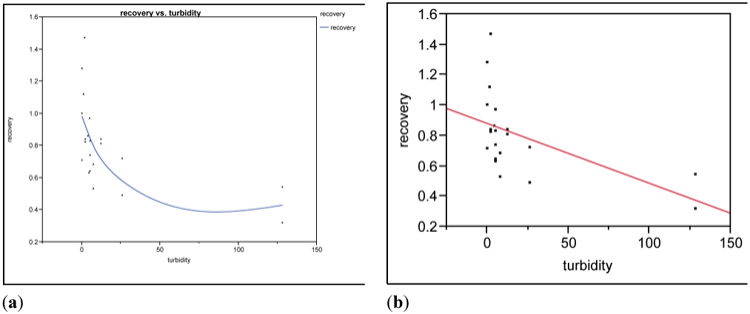
UF recovery efficiency by turbidity, for ΦX174, with spline (**a**); and linear regression line overlaid (**b**).

In general, water quality parameters were not observed in the present study to be associated with UF recovery efficiencies. The only exception was a negative correlation between ΦX174 recovery and turbidity {[corr = −0.5473, 95% CI = (−0.7873, −0.1634)]Figure 2}. Recovery efficiencies of E1, MS2 and *C. parvum* also declined as turbidity increased, up to 26 NTU. However, when 128-NTU experiments were included in the analyses there was no significant correlation between turbidity and recovery efficiency for these microbes. Taken together, the average recovery data indicate that UF achieved effective microbial recovery for all the study microbes and that it is robust enough to handle a diverse set of water matrices.

While the results of this study demonstrated that viruses could be effectively recovered from environmental water samples using the UF procedure, it was difficult to discern whether turbidity negatively affected the performance of the Centricon and PEG procedures or had an adverse impact on the cell culture assay because the cell culture assays were sensitive to toxicity and cell culture health. Some degree of cell toxicity was observed for many of the water samples, and plaque counts were often obtained from dilutions. However, toxicity was not observed to a greater extent in the more turbid water samples. E1 recovery efficiency with PEG was positively correlated with specific conductance [corr = 0.7740, CI = (0.5230, 0.9014)], which suggests that PEG precipitation might be a better method for ground water samples than the Centricon procedure since ground water tends to have higher ionic content.

Although cell toxicity was not observed at a higher frequency as turbidity increased, average PEG recoveries for E1 from Chattahoochee river high turbidity water were much lower than for other water types. The reason for such low PEG recoveries from this sample are unknown, but could have been due in part to a smaller percentage of the final sample volume being assayed. The low recoveries could also have been due to constituents in the high turbidity water that negatively impacted the plaque assay without resulting in overt cell toxicity. Average E1 PEG recoveries from Jefferson City ground water were much higher than the average recovery for other water types. However, there were no inconsistencies in any of the measured sample parameters (UF concentrate “*output*,” PEG final volume) that would explain such high recovery rates.

When data from all three viruses were combined, recovery efficiencies for the secondary concentration procedures were consistently higher with PEG precipitation than the Centricon procedure (*p* = 0.03). Centricon Plus-70 concentrators are simple to use and do not require special equipment other than a standard centrifuge. However, they are susceptible to clogging and the sample chamber is limited to a 70-mL sample volume. For volumes larger than 70 mL, two Centricon units can be used and the retentates combined, as was done in this study, or they can be double- or triple-loaded. However, Centricon Plus-70 units are relatively expensive, making use of multiple units per sample a potentially unsustainable sample processing approach. The manufacturer's recommended spin time is 15–40 min, but it often took upwards of 60 min to process the water concentrates for this study. During three experiments, only 75–80 mL lake water concentrates could be processed due to filter clogging. Interestingly, the turbidity levels of these waters were 4–5 NTU, and none of the other water quality characteristics were unusual. The recovery efficiencies for these samples were not lower than average, which suggests that filter clogging does not necessarily lead to poorer recoveries. PEG precipitation allows for the potential to process a substantial volume of water, although volumes were limited to 150 mL in the experimental design for this study. A high speed centrifuge capable of reaching 10,000 × *g* is typically recommended to sediment virus precipitates, especially in 150-mL samples, which may prohibit some laboratories from performing this method.

Average *C. parvum* recovery efficiency after IMS was 95% and the total method recovery efficiency averaged 84%. Other researchers have reported average recoveries of *C. parvum* from ground water and surface water using hollow-fiber tangential flow UF followed by IMS to be 14%–74% [1,9,17]. With the exception of a 13% recovery efficiency for one experiment, recovery of *C. parvum* by UF and IMS was ≥40% in the present study. This is further evidence suggesting that tangential-flow hollow-fiber UF can be an effective alternative to microfiltration in conjunction with USEPA Method 1623 procedures [23]. Iron [26] and turbidity [27] have been identified as factors impacting performance of IMS, though other researchers have not observed these relationships [1,9,25]. In this study, no correlations were observed between IMS performance and iron concentration or turbidity.

While the recovery data from this study was generated using tangential-flow hollow-fiber UF, dead-end ultrafiltration (DEUF) has also been used for concentration of large volume surface water and ground water samples [31,32]. Tangential-flow UF may be less susceptible to filter clogging than DEUF because of the scouring effect from recirculating water through the ultrafilter cartridge, which increases the tendency to keep particles suspended in the recirculating water sample versus clogging filter pores. However, DEUF requires less operator training and is readily field-deployable. While no published side-by-side data are available for comparing recovery efficiencies between tangential-flow UF and DEUF, comparison of the data from this study with previous work using DEUF for lake water [32] indicate that recovery rates of tangential-flow UF and DEUF are similar.

This study was associated with several limitations. First, the study focused on measuring UF performance using culture and microscopy analyses, and did not incorporate use of molecular testing (e.g., real-time PCR). This was due primarily to an interest in evaluating the effects of turbidity and other water quality parameters on microbial recovery efficiency, which is more directly addressed through the use of direct measure techniques such as quantitative culture and microscopy. Other studies have demonstrated that molecular assays can be effectively performed on UF concentrates [32,33]. Another limitation of this study was the large gap in turbidity between 26 NTU and 128 NTU, which required interpolation of values over a large data gap, potentially affecting the ability of the statistical test to find significance associated with turbidity.

## 5. Conclusions

Average UF recovery efficiencies for the six enteric microbes used in this study were 66%–95%. Average recovery efficiencies for the secondary concentration methods for *C. parvum* and the viruses ranged from 35% to 95%. Most of the measured water quality parameters were not significantly associated with UF recovery efficiencies, with one exception. Recovery of ΦX174 was negatively correlated with turbidity. The data from this study demonstrate that the hollow-fiber UF procedure, alone or in conjunction with secondary processing methods, can be an efficient and robust technique for recovering a diverse array of microbes, including viruses, bacteria, bacterial spores, and protozoan parasite oocysts from ground water and surface water samples.

## Figures and Tables

**Table 1:** Water quality data for environmental water samples.

Site	pH	Turbidity (NTU)	Specific Conductance (μS/cm at 25 °C)	Hardness (mg/L as CaCO_3_)	Alkalinity (mg/L as CaCO_3_)	Total iron (mg/L Fe)	Total Suspended Solids (TSS) (mg/L as C)	Dissolved Organic Carbon (DOC) (mg/L as C)	Total Suspended Solids (TSS) (mg/L)
	5.4	128	53.0	16	14	1.6	5.5	2.0	57
Chattahoochee river	6.1	26.0	66.0	17	16	0.75	4.3	2.6	NT *
	6.9	5.25	61.0	16	16	0.24	1.8	1.4	7.2

	7.7	12.4	89.0	26	25	0.59	7.9	6.0	13
Murphy Candler lake	6.7	7.58	103	31	31	0.69	10	9.2	5.6
	7.6	4.09	119	28	27	0.82	12	13	4.0

	8.2	0.097	233	120	124	0.08	7.0	7.3	0.3
Lawrenceville ground water	8.3	0.113	235	110	96	0.09	10	9.9	0.15
	8.2	0.135	237	110	99	0.07	11	11	<0.01

	8.1	2.11	693	290	250	<0.02	28	20	2.4
Jefferson Cityground water	7.8	1.89	572	290	240	<0.02	28	28	1.7
	7.7	1.20	573	300	250	0.02	28	25	0.8

	9.2	5.25	70.5	22	24	0.08	7.7	0.90	6.5
Allatoona lake	9.0	4.98	73.1	21	22	0.07	NT *	NT *	7.2
	9.6	4.55	70.0	24	25	0.06	NT *	NT *	8.0

Note:

* Not tested.

**Table 2:** Ultrafiltration (UF) recovery efficiencies for study microbes.

Site	*n*	Average % Recovery Efficiency (SD)					

		ΦX174	MS2	Echovirus 1	*E. coli*	*C. perfringens*	*C. parvum*
Chattahoochee river	6 *	58 (16)	91 (38)	69 (9)	98 (11)	86 (15)	78 (33)
Murphy Candler lake	5	74 (14)	65 (33)	50 (15)	85 (38)	55 (10)	70 (24)
Lawrenceville	4 †	100 (23)	85 (23)	130 (24)	ND ‡	73 (21)	120 (37)
Jefferson City	4	110 (31)	77 (8)	45 (27)	87 (16)	69 (10)	120 (44)
Allatoona lake	3 §	81 (17)	53 (19)	53 (32)	79 (12)	100 (9)	100 (11)

Cross-site avg.		81 (26)	76 (29)	66 (33)	88 (23)	75 (20)	95 (37)

Notes:

* *n* = 4 for *E. coli*;

† *n* = 3 for echovirus 1;

‡ No data because *E. coli* were not present at sufficient concentrations for recovery efficiency calculation;

§ *n* = 2 for *C. perfringens*.

**Table 3:** Secondary concentration recovery efficiencies.

Site	*n*	Average % bRecovery Efficiency (SD)						
		ΦX174		MS2		Echovirus 1		*C. parvum*
			
		Centricon	PEG	Centricon	PEG	Centricon	PEG	IMS
Chattahoochee river	6	70 (27)	88 (18)	81 (16)	69 (19)	26 (28)	52 (43)	109 (56)
Murphy Candler	5	67 (13)	63 (14)	81 (39)	73 (12)	30 (12)	60 (41)	87 (16)
Lawrenceville	4 *	80 (10)	81 (11)	93 (13)	99 (28)	56 (18)	89 (22)	85 (27)
Jefferson City	4 *	69 (6)	72 (5)	81 (8)	75 (11)	39 (19)	220 (100)	110 (47)
Lake Allatoona	3 †	64 (19)	73 (20)	55 (13)	81 (9)	31 (9)	64 (21)	48

Cross-site avg.		70 (17)	76 (16)	79 (23)	78 (19)	35 (21)	92 (80)	95 (40)

Notes:

* *n* = 3 for *C. parvum*;

† *n* = 1 for *C. parvum*.

## Appendix

**Table A1:** Overall recovery efficiencies for microbes undergoing secondary concentration.

Site	*n*	Overall % Recovery Efficiency (SD)						

		ΦX174		MS2		Echovirus 1		*C. parvum*
			
		Centricon	PEG	Centricon	PEG	Centricon	PEG	IMS
Chattahoochee river	6	41 (21)	49 (14)	69 (19)	66 (36)	17 (17)	34 (28)	82 (43)
Murphy Candler	5	50 (14)	46 (12)	56 (33)	44 (16)	15 (7)	32 (26)	60 (21)
Lawrenceville	4 *	80 (24)	83 (28)	77 (13)	80 (7)	60 (13)	100 (21)	117 (43)
Jefferson City	4 †	73 (20)	76 (21)	62 (8)	58 (9)	15 (7)	77 (21)	109 (12)
Lake Allatoona	3 ‡	53 (23)	61 (29)	27 (4)	42 (13)	15 (8)	30 (9)	47

Cross-site avg.		58 (24)	61 (23)	61 (24)	59 (24)	22 (19)	51 (35)	84 (38)

Notes:

* *n* = 3 for *C. parvum* and Echovirus 1;

† *n* = 1 for *C. parvum*;

‡ *n* = 3 for *C. parvum*.
